# Tight lower bounds for block-structured integer programs

**DOI:** 10.1007/s10107-025-02296-z

**Published:** 2025-10-27

**Authors:** Christoph Hunkenschröder, Kim-Manuel Klein, Martin Koutecký, Alexandra Lassota, Asaf Levin

**Affiliations:** 1 (Formerly) Institut für Mathematik, TU Berlin, Berlin, Germany; 2 Institute for Theoretical Computer Science, University of Lübeck, Lübeck, Germany; 3 Computer Science Institute, Charles University, Prague, Czech Republic; 4 Eindhoven University of Technology, Eindhoven, The Netherlands; 5 Faculty of Data and Decisions Sciences, The Technion, Haifa, Israel

**Keywords:** Integer programming, *n*-fold IPs, Tree-fold IPs, Multi-stage IPs, (Unconditional) lower bounds, ETH, Subset sum, 90C10, 68Q27

## Abstract

We study fundamental block-structured integer programs called tree-fold and multi-stage IPs. Tree-fold IPs have a constraint matrix with independent blocks linked together by few constraints in a recursive pattern. Transposing this constraint matrix yields the constraint matrix of multi-stage IPs. The state-of-the-art algorithms to solve these IPs have an exponential gap in their running times, making it natural to ask whether this gap is inherent. We answer this question in the affirmative. Assuming the Exponential Time Hypothesis, we prove lower bounds showing that the exponential difference is necessary. This also proves that the known algorithms are essentially optimal. Moreover, we prove unconditional lower bounds on the size of the Graver basis elements, a fundamental building block of all known algorithms to solve these IPs. This shows that none of the current approaches can be improved beyond this bound unconditionally.

## Introduction

In the past years, there has been tremendous progress in the algorithmic theory and for the applications of *block-structured integer programming*. An *integer program (IP)* in standard form is the problem $\min \{c^\intercal x: \ Ax = b,\, l \le x \le u,\, x \in \mathbb {Z}^n\}$. We deal with the setting where the constraint matrix *A* exhibits a certain block structure. One of the most prominent block-structure families is that of *n-fold* integer programs (*n*-fold IPs), in which the constraint matrix *A* decomposes into a block-diagonal matrix after the first few rows are deleted. In other words, *n*-fold IPs are constructed from independent IPs (that are IPs that do not share any variables) of small dimensions that are linked by a few constraints. The generalization, in which the diagonal blocks themselves have an *n*-fold structure recursively, is called *tree-fold* IPs. The transpose of a tree-fold matrix yields another class of highly relevant constraint matrices called *multi-stage* matrix. We formally define these families of matrices next.

### Definition 1

(*Tree-fold and multi-stage matrices*) Any matrix $A \in \mathbb {Z}^{m \times n}$ is a tree-fold matrix with one level and level size *m*. A matrix *A* is a tree-fold matrix with $\tau \ge 2$ levels and level sizes $\sigma = (\sigma _1, \dots , \sigma _\tau ) \in \mathbb {Z}_{\ge 1}^{\tau }$ if deleting the first $\sigma _1$ rows of *A* decomposes the matrix into a block-diagonal matrix, where each block is a tree-fold matrix with $\tau -1$ levels and level sizes $(\sigma _2, \dots , \sigma _\tau )$.

If $A^\intercal$ is a tree-fold matrix with $\tau$ levels and level sizes $\sigma$, then *A* is called a *multi-stage matrix* with $\tau$ stages and stage sizes $\sigma$.

**Fig. 1:**
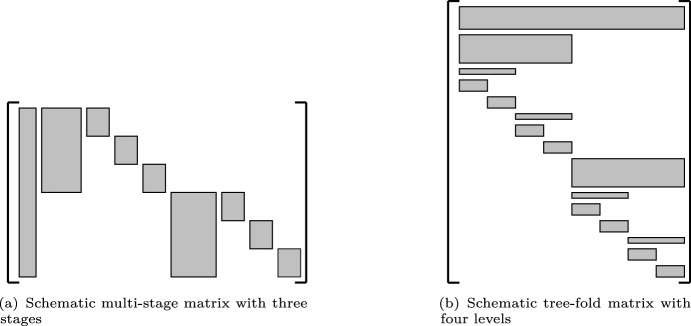
The left figure shows a schematic multi-stage matrix with three stages. The right figure shows a schematic tree-fold matrix with four levels. All entries within a gray rectangle can be non-zero, and all entries outside the rectangles must be zero

For a schematic picture, see Figure 1.

### Definition 2

(*n*-*fold and* 2-*stage stochastic matrices*) The special case of a tree-fold matrix with two levels is called *n*-fold. Respectively, a multi-stage matrix with two stages is called 2-stage stochastic matrix.

**Fig. 2:**
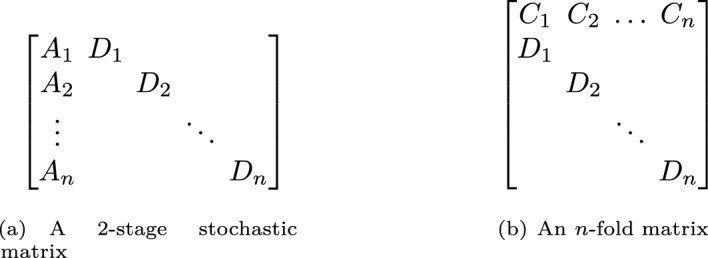
The left figure shows a 2-stage stochastic matrix with blocks $A_i$ and $D_i$, $i = 1, 2, \dots , n$. The right figure shows an *n*-fold matrix with blocks $C_i$ and $D_i$, $i = 1, 2, \dots , n$. All entries not belonging to a block are zero

For a picture, see Figure 2. We refer to the $A_i$, or respectively, $C_i$ blocks as the global part of the constraint matrices as these few variables, or respectively, few constraints affect all diagonal blocks $D_i$. We refer to the diagonal blocks $D_i$ as local.

The study of *n*-fold IPs was initiated in [33]. A milestone was the fixed-parameter tractable algorithm by Hemmecke et al. [14] whose running time depends polynomially on the dimension *n*, and exponentially only on the sizes of the small blocks. Faster and more generally applicable algorithms were subsequently developed, including strongly polynomial time algorithms, near-linear (in *n*) time algorithms [4, 8, 9, 20, 30, 33], and a new result where entries in the global part can be large [6]. At the same time, *n*-fold IPs found many applications, for instance in scheduling problems, stringology, graph problems, and computational social choice, see e.g. [3, 12, 15, 18, 19, 24–28], solving long-standing open problems.

Multi-stage IPs and their special case for $\tau = 2$ (2-stage stochastic IPs) have been studied even longer than *n*-fold IPs, going back to the work of Aschenbrenner and Hemmecke [2]. They are commonly used in stochastic programming and often used in practice to model uncertainty of decision making over time [1, 7, 21, 31]. The first known upper bounds on the complexity of solving multi-stage IPs had a huge and non-explicit dependence on $\tau$ and $\sigma$ in their running time. The upper bound was subsequently improved to have an exponential tower of height $\tau$ with $\Vert \sigma \Vert _1$ appearing at the top (multiplied by a polynomial in the encoding length of the input *I*), and only very recently to have a triple exponential (in $\Vert \sigma \Vert _1$) running time (multiplied by $|I|^{O(1)}$) [5, 9, 22, 23]. Note that the encoding length of an instance is linear in $\sigma$, $\tau$, *n*, *m*, and the largest entry in the constraint matrix, the right-hand side *b* and the objective function *c*. In [6], a new FPT time algorithm to decide feasibility of a 2-stage stochastic IP is presented that can also handle large entries in the global part (i.e., the largest entry in the global part is not a parameter). Very recently Eisenbrand and Rothvoss [10] generalized this result to handle 2-stage stochastic IP also with an objective function.

Intriguingly, the algorithms for tree-fold IPs have a running time that depends only double-exponentially on $\Vert \sigma \Vert _1$ [9]. A natural response to seeing this exponential gap is to ask whether it is inherent, and whether multi-stage integer programming is indeed harder than tree-fold IPs despite their similar nature of constraint matrices. This question was partially answered when Jansen et al. [17] showed that, assuming the Exponential Time Hypothesis (ETH), 2-stage stochastic IPs require a double-exponential running time in $\Vert \sigma \Vert _1$. This contrasts with known single-exponential algorithms with respect to $\Vert \sigma \Vert _1$ for *n*-fold IPs. Also for tree-fold IPs, a double-exponential lower bound with respect to $\tau$ is known [29], although this is stated in terms of the parameter *tree-depth*, which is linked to the largest number of non-zeroes in any column (see Section 6), still leaving the exact dependence on the number of levels $\tau$ open.

We prove running time lower bounds of all aforementioned block-structured integer programs, answer the question whether the exponential gap is necessary in the affirmative assuming ETH (see Conjecture 1), and (nearly) close the gaps between algorithms and lower bounds:

1. (**Theorem** 2) We show an ETH-based lower bound for multi-stage IPs that is triple exponential in the number of stages $\tau$ even when the stage sizes $\sigma$ are constant, and recover the existing double-exponential lower bound (in $\Vert \sigma \Vert _1$) [17] as a special case. This lower bound is comparable to the running time of the currently fastest algorithm [23].
2. (**Theorem** 3) We show an ETH-based lower bound for tree-fold IPs which recovers as a special case the result of [29], and is comparable to the running time of the currently best known algorithm [9].
3. (**Corollary** 3) A particularly interesting consequence of Theorem 3 is a lower bound of $2^{o(\sigma _1 \sigma _2)}$ on the parameters for the special class of *n*-fold IPs.

The core technical idea behind the lower bounds in this paper relates *k*-diagonal matrices to block-structured matrices. A *k*-diagonal matrix is a matrix that has non-zero entries only for those entries which are close to and to the right of the diagonal line of the matrix (i.e. the difference of the indices *i*, *j* of a non-zero entry $A_{i,j}$ must be $0 \le j-i \le k$), see Section 2 for a formal definition.

4. (**Theorem** 1 and **Lemma** 1) Every *k*-diagonal matrix can be reordered to obtain a multi-stage or a tree-fold matrix.

We believe this result is of independent interest, as it provides a new tool for solving combinatorial problems: Formulating any problem as a matrix with constant bandwidth is enough to be able to apply the tree-fold or multi-stage integer programming algorithms to solve it efficiently.

We construct hard instances that have bi- or tri-diagonal structure, which yields the required lower bounds. For the multi-stage IPs lower bound, we combine this idea with splitting one complicated constraint carefully into several sparse constraints with only few variables, similarly to the well-known reduction from a 3-Sat formula to a 3-Sat instance where each variable only appears constantly often. This is done in Section 3. Section 4 continues with the lower bound for tree-fold IPs.

The central concept to all algorithms solving these block-structured integer programs is the *Graver basis* $\mathcal {G}(A)$ of the constraint matrix *A*, or in case of the new result [6], of some reduced constraint matrix $A'$ with block-structure and small entries. A closer examination shows that the main factor driving the complexity of those algorithms are the $\ell _\infty$- and $\ell _1$-norms of elements of $\mathcal {G}(A)$ ($\mathcal {G}(A')$). Asymptotical improvements on the bound of the norm of the Graver elements would lead to improved algorithms, contradicting ETH. In the following, we show lower bounds on the norm of elements of $\mathcal {G}(A)$ for block-structured matrices which are unconditional, i.e. do not rely on the assumption of ETH.

We demonstrate these norm lower bounds on the matrices used in the proofs of Theorems 2 and 3, as described in Section 5. Our unconditional lower bounds for Graver basis elements trace back to the matrix hardness that was proven for 2-stage stochastic IPs in [22]. Extending these results to multi-stage IPs and advancing to tree-fold and *n*-fold IPs though required the here presented concept of rearranging bi- and tri-diagonal matrices. In Section 6, we briefly express our work in terms of the parameter *tree-depth* that is also commonly used to describe block-structured integer programs.

This paper partially builds on an arXiv preprint [9]. The present paper provides stronger and novel results. Specifically, it introduces bi- and tri-diagonal matrices formally as crucial components for the hardness proofs. This approach enables us to reframe the results for tree-fold IPs in terms of *levels* and the maximum number of rows in a level, rather than stating them solely on tree-depth as in both [9, 29], which is the product of these parameters. This refined perspective allows for a more nuanced analysis of *n*-fold IPs which derives at its (near tight) lower bound, which was unattainable with previous methods. In the context of multi-stage IPs, our investigation picks up from where the proof in [17] for 2-stage stochastic IPs concluded. Notably, the proof for 2-stage stochastic IPs did not involve or observe any potential rearrangement of the stages; the first stage comprised only one variable, and the 2-stage stochastic structure emerged naturally from the underlying problem. The here presented results are only possible due to the insights into bi- and tri-diagonal matrices.

## About *k*-diagonal matrices

This section is devoted to matrices which only have non-zero entries along a tube on the diagonal. We call such matrices *k*-diagonal, and define them formally as:

### Definition 3

(*k*-*diagonal Matrix*) A matrix $\mathcal {A}\in \mathbb {Z}^{m \times n}$ is *k*-*diagonal* if $a_{i,j} = 0$ for $i \notin \{j,j+1, \dots , j+k-1\}$, $i = 1, 2, \dots , m$ and $j = 1, 2, \dots n$, where $a_{i,j}$ is the entry of matrix $\mathcal {A}$ in row *i* and column *j*.^1^

In this paper, we write $\mathcal {A}$ to refer to matrices with a specific structure that will be clear from the context. To prove the desired lower bounds on the block-structured IPs, we are particularly interested in 2- and 3-diagonal matrices which we also refer to as bi- and respectively tri-diagonal matrices. It follows from the definition above that they have the following structure:

### Definition 4

(*Bi-diagonal, Tri-diagonal Matrix*) Bi- and tri-diagonal matrices are special cases of *k*-diagonal matrices: A matrix $\mathcal {A}\in \mathbb {Z}^{m \times n}$ is *bi-diagonal* (2-diagonal) if $a_{i,j} = 0$ for $i \notin \{j,j+1\}$, $i = 1, 2, \dots , m$ and $j = 1, 2, \dots n$. A matrix $\mathcal {A}\in \mathbb {Z}^{m \times n}$ is *tri-diagonal* (3-diagonal) if $a_{i,j} = 0$ for $i \notin \{j,j+1,j+2\}$, $i = 1, 2, \dots , m$ and $j = 1, 2, \dots n$.

We would like to briefly mention that our definition of *k*-diagonal matrices differs slightly from other common definitions of *k*-diagonal matrices, where the entries of the matrices are centered around the diagonal line and not only on the right of the diagonal line like in our definition.

We show that any *k*-diagonal matrix can be viewed as a multi-stage matrix, and the transpose of any *k*-diagonal matrix can be viewed as a tree-fold matrix. In particular, we can rearrange a bi- or tri-diagonal matrix *A* to a multi-stage or tree-fold matrix with the desired parameters to prove our lower bounds.

While the next theorem requires quite specific matrix dimensions, note that any *k*-diagonal matrix can be extended to the desired matrix dimensions while remaining *k*-diagonal by adding all-zero rows or columns.

### Theorem 1

Let $\tau \ge 1$ and $k \ge 2$, $\sigma \in \mathbb {Z}_{\ge 1}^\tau$, and define $S \mathrel {\mathop :}=\prod _{i=1}^\tau (\sigma _i + 1)$. Let $\mathcal {A}\in \mathbb {Z}^{ (k-1)S \times ((k-1)S - (k-1))}$ be *k*-diagonal. Then $\mathcal {A}$ is a multi-stage matrix with $\tau$ stages and stage sizes $(k-1)\sigma$, up to column permutations.

### Proof idea 1

(of Theorem 1) Observe that if we delete sufficiently many consecutive columns in a *k*-diagonal matrix, we split the matrix into two independent blocks. Doing this recursively to the independent blocks yields the desired multi-stage matrix.

### Proof of Theorem 1

The proof is by induction on $\tau$, and the base case for $\tau =1$ is trivial as any matrix can be interpreted as a multi-stage matrix of just one stage.

**Fig. 3:**
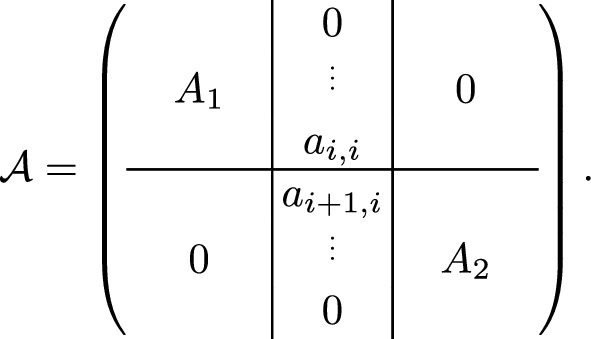
Displayed is a bi-diagonal (2-diagonal) matrix, and a column *i* that is separating the matrix into two independent bi-diagonal matrices $A_1$ and $A_2$

For $\tau > 1$, observe that if we delete $k-1$ consecutive columns $i, i+1, \dots , i+k-2$, we can partition the remaining matrix into two blocks $$\left( {\begin{array}{c}A_1\\ 0\end{array}}\right) $$, $$\left( {\begin{array}{c}0\\ A_2\end{array}}\right) $$ whose columns are orthogonal to each other. An example is depicted in Figure 3 for $k=2$.

**Fig. 4:**
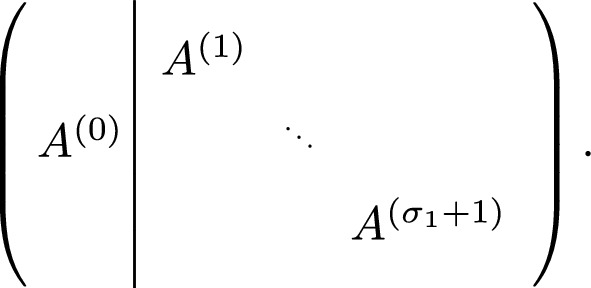
Rearranging the columns of $\mathcal {A}$ to obtain a 2-stage stochastic constraint matrix where each $A^{(i)}$ for $i \ge 1$ is *k*-diagonal and independent

Let $$S' \mathrel {\mathop :}=\prod _{i=2}^\tau (\sigma _i + 1)$$. Define

$$ L \mathrel {\mathop :}=\bigcup _{i=1}^{\sigma _1} \{(k-1) i S^\prime - k + 2, (k-1) i S^\prime - k + 1, \dots , (k-1)iS^\prime \} $$

and let $A^{(0)} \in \mathbb {Z}^{(k-1)S \times (k-1)\sigma _1}$ comprise the columns with indices in *L*. $A^{(0)}$ is the matrix containing all columns that upon deletion, separate the instance *A* into $\sigma _1 + 1$ independent *k*-diagonal submatrices. In other words, permuting the columns in $A^{(0)}$ to the front, we obtain a 2-stage stochastic matrix with stage sizes $((k-1)\sigma _1,S^\prime )$ and $\sigma _1 +1$ second stage matrices, see Figure 4 for a schematic picture. We call these (second stage matrices) submatrices $A^{(\ell )}$ for $\ell =1,\dots ,\sigma _1+1$.

The matrix $A^{(\ell )} \in \mathbb {Z}^{(k-1)S^\prime \times (k-1)(S^\prime -1)}$ arises from $\mathcal {A}$ (up to column permutation) by restricting to row indices *i* with $a \le i \le b$ with $a = (k-1)(\ell -1)S^\prime +1$ and $b = (k-1)\ell S^\prime$ and column indices *j* with $a \le j \le b$ with $a = (k-1)(\ell -1)S^\prime + 1$ and $b = (k-1) (\ell -1) S^\prime +1 + (k-1)S^\prime -1 -(k-1) = (k-1) \ell S^\prime - (k-1)$.

The matrices are independent as $k-1$ columns with respect to the ordering of the original *k*-diagonal matrix have been removed (shifted to the front) between each consecutive pair of submatrices.

By induction, each $A^{(\ell )}$ is a multi-stage matrix with $\tau - 1$ stages and stage sizes $((k-1)\sigma _2,\dots ,(k-1)\sigma _{\tau })$, after a suitable permutation. $\square$

Observing that multi-stage matrices and tree-fold matrices are the transpose of each other, we can easily show the following lemma:

### Lemma 1

Let $\tau \ge 1$, $\sigma \in \mathbb {Z}_{\ge 1}^\tau$, and define $S \mathrel {\mathop :}=\prod _{i=1}^\tau (\sigma _i + 1)$. Let $\mathcal {A}^\intercal \in \mathbb {Z}^{((k-1)S) \times ((k-1)S - (k-1))}$ be *k*-diagonal. Then $\mathcal {A}$ is a tree-fold matrix with $\tau$ levels and level sizes $(k-1) \sigma$, up to row permutations.

### Proof

We can apply Theorem 1 to $\mathcal {A}^\intercal$ to obtain the rearrangement yielding a multi-stage matrix $\mathcal {M}$ with $\tau$ stages and stage sizes $(k-1) \sigma$. Taking the transpose of $\mathcal {M}$ yields a tree-fold matrix with the desired parameters. $\square$

The following statements for bi- and tri-diagonal matrices immediately follow as special cases of Theorem 1 and Lemma 1:

### Corollary 1

Let $\tau \ge 1$, $\sigma \in \mathbb {Z}_{\ge 1}^\tau$, and define $S \mathrel {\mathop :}=\prod _{i=1}^\tau (\sigma _i + 1)$.

*i*): Let $\mathcal {A}\in \mathbb {Z}^{ S \times (S - 1)}$ be bi-diagonal. Then $\mathcal {A}$ is a multi-stage matrix with $\tau$ stages and stage sizes $\sigma$, up to column permutations.
*ii*): Let $\mathcal {A}\in \mathbb {Z}^{2S \times (2S - 2)}$ be tri-diagonal. Then $\mathcal {A}$ is a multi-stage matrix with $\tau$ stages and stage sizes $2\sigma$, up to column permutations.

### Corollary 2

Let $\tau \ge 1$, $\sigma \in \mathbb {Z}_{\ge 1}^\tau$, and define $S \mathrel {\mathop :}=\prod _{i=1}^\tau (\sigma _i + 1)$.

*i*): Let $\mathcal {A}^\intercal \in \mathbb {Z}^{ S \times (S - 1)}$ be bi-diagonal. Then $\mathcal {A}$ is a tree-fold matrix with $\tau$ levels and level sizes $\sigma$, up to row permutations.
*ii*): Let $\mathcal {A}^\intercal \in \mathbb {Z}^{2S \times 2S - 2}$ be tri-diagonal. Then $\mathcal {A}$ is a tree-fold matrix with $\tau$ levels and level sizes $2\sigma$, up to row permutations.

Note that if $\mathcal {A}$ itself is *k*-diagonal, we can add $k-1$ all zero columns in the front. This way we obtain a matrix $\tilde{A}$ for which $\tilde{A}^{\intercal }$ is *k*-diagonal.

## Multi-stage integer programming

This section presents our main result regarding the hardness of multi-stage IPs. In particular, we reduce 3-Sat to multi-stage IPs.

### Conjecture 1

(ETH [16]) The 3-Sat problem cannot be solved in time $2^{o(N)}$, where *N* is the number of variables contained in the formula.

### Theorem 2

(A lower bound for multi-stage IPs) For every number of stages $\tau \ge 1$, there is a family of multi-stage IPs with $\tau$ levels, constant stage sizes, and entries bounded by a constant, such that there is no algorithm solving every instance *I* of this family of multi-stage IPs in time $2^{2^{2^{o(\tau )}}} |I|^{O(1)}$ unless ETH fails.

By considering multi-stage IPs with constant stage sizes, we immediately get that every algorithm has to have a triple exponential dependency on $\tau$ when parametrized by the number of stages $\tau$ and the largest value of any coefficient of *A*. Thus, we cannot increase the dependency on the other parameters to decrease the dependency on $\tau$. Specifically, we rule out any algorithm solving multi-stage IPs in time less than triple exponential in $\tau$ for the parameters $\tau , \sigma , \Delta , \Vert c\Vert _{\infty }$, $\Vert b\Vert _{\infty },\Vert \ell \Vert _{\infty }$ where $\Delta$ is the largest absolute value in the constraint matrix. This proves tightness of the triple exponential complexity with respect to $\tau$ of the current state-of-the-art algorithm of Klein and Reuter [23].

### Proof idea 2

(of Theorem 2) The proof starts where the proof for the double exponential lower bound in [17] ends. Specifically, we are given a 2-stage stochastic matrix which is, under ETH, the double exponentially hard instance for 2-stage stochastic IPs.

The blocks of the second stage, that is, the small blocks arranged along the diagonal are each *nearly* a diagonal matrix. That is, they are a diagonal matrix but with an extra row. Note that this row corresponds to a summation of scaled summands, i.e., it corresponds to $az_1+bz_2+cz_3+dz_4+\dots$ for row entries $a, b, \dots$ and variables $z_1, z_2, \dots$, respectively.

This sum is equal to $s_2 + cz_3 + dz_4+\dots$ with $az_1+bz_2 = s_2$ which, by repeated application, yields a bi-diagonal matrix. Handling the few extra rows finally gives us the desired form to apply Corollary 1 to each of the second stage matrices giving us a multi-stage IP. We finally apply the whole reduction to prove that such a running time would violate ETH.

### Proof of Theorem 2

For $x \in \mathbb {Z}_{\ge 0}$ and $\eta = \lceil \log _2(x + 1) \rceil + 1$, let ${{\,\textrm{enc}\,}}(x) \in \mathbb {Z}^{1 \times \eta }$ denote the binary encoding of *x*, i.e., $x = \sum _{i=0}^{\eta -1} 2^{i}({{\,\textrm{enc}\,}}(x))_{i+1}$ with $({{\,\textrm{enc}\,}}(x))_{i+1} \in \{0,1\}$ denoting the *i*-th bit.

In [17], the 3-Sat problem with *N* variables and *O*(*N*) clauses is reduced to a 2-stage stochastic IP of the form 
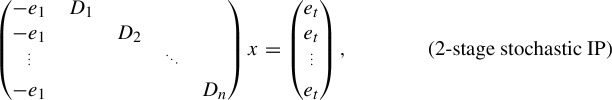
 where $e_i$ is the *i*-th canonical unit vector, *t* is the number of rows in the $D_i$ matrix being of shape1where *E* is called the *encoding matrix*. Here, $x_i, y_i, q_i \in \mathbb {Z}_{\ge 0}$ are numbers generated by reducing a 3-SAT formula to 2-stage stochastic IPs, see [17]. All variables have a lower bound 0, an upper bound which is $O(2^{O(N^2\log _2(N))})$, and the objective function is zero, i.e., only feasibility is sought.

There are $n \in O (N^2)$ blocks $D_i \in \mathbb {Z}^{t \times s}$ with $s = t + 1 \in O(\log _2(N))$, all coefficients are bounded by 2 in absolute value, and the first stage size is $\sigma _1 = 1$.

For $E \in \mathbb {Z}^{\eta \times (\eta + 1)}$, let $E^\dagger$ denote the matrix arising from *E* by reversing the order of the rows and columns, i.e., $E^\dagger _{i,j} = E_{\eta - i + 1, \eta - j + 2}$. In particular, it has the following form:Similarly, let ${{\,\textrm{enc}\,}}^\dagger (x)$ arise from ${{\,\textrm{enc}\,}}(x)$ by reversing the order of the entries. Hence, by permuting rows and columns and inserting a zero row, a single block can be brought into the shapewhere $c^{(k)} \mathrel {\mathop :}=({{\,\textrm{enc}\,}}(q_k), {{\,\textrm{enc}\,}}^\dagger (x_k), {{\,\textrm{enc}\,}}(y_k))$ concatenates the bit encodings, and $B^\intercal$ is a bi-diagonal matrix, i.e.,

2 $$\begin{aligned} B_{i,j}&= 0 \text { for } i \notin \{j,j-1\}. \end{aligned}$$

In the next step, similar to the approach in [9], we replace ${ c^{(k)} \atopwithdelims ()B}$ by a tri-diagonal matrix $T_k$.

Fix an index *k* and denote ${c \atopwithdelims ()B} \mathrel {\mathop :}={c^{(k)} \atopwithdelims ()B}$ for short. Denote the global variable of (2-stage stochastic IP) by *r*, the variables of the diagonal block $D_k$ by $z \mathrel {\mathop :}=z^{(k)}$ and consider the topmost constraint $-r + cz = 0 \Leftrightarrow \sum _{i=1}^{t+1} c_i z_i = r$ of this block. We introduce new variables $p \in \mathbb {Z}^{t+1}$ and constraints as follows:

$$\begin{aligned} p_1&= - r \\ p_{i+1} - p_{i}&= c_i z_i \hspace{3cm} i=1,\dots ,t \\ -p_{t+1}&= c_{t+1} z_{t+1}. \end{aligned}$$

Summing up all the equations, we retrieve $\sum _{i=1}^{t+1} c_iz_i = r$. Alternating the variables $p_i$ and $z_i$, we can replace the constraint $\sum _{i=1}^{t+1} c_i z_i = r$ with the systemWe call this new system $e_1 r + \tilde{S} \tilde{z} = 0$, where $\tilde{S}$ is the constraint matrix without the first column, and $\tilde{z}$ is the corresponding solution vector. Formally, we have

$$ \tilde{S}_{i,j} \mathrel {\mathop :}={\left\{ \begin{array}{ll} 1 &  j = 2i - 1, \\ -c_{i-1} &  j = 2i - 2, \\ -1 &  j = 2i - 3, \\ 0 &  \text {else}, \end{array}\right. } \hspace{40pt} \text {for } \left\{ \begin{array}{l} i = 1,\dots , t+2, \\ j = 1,\dots , 2t+2. \end{array} \right. $$

In particular, this definition implies

3 $$\begin{aligned} \tilde{S}_{i,j}&= 0 \quad \text { for } \quad i \notin \left\{ \tfrac{j+1}{2}, \tfrac{j+2}{2}, \tfrac{j+3}{2}\right\} . \end{aligned}$$

We obtain $\tilde{B}$ from *B* accordingly by adding zero columns corresponding to the new variables *p*, and adding one zero row on the top. Formally we define

$$\begin{aligned} \tilde{B}_{i,j}&\mathrel {\mathop :}={\left\{ \begin{array}{ll} B_{i-1,j/2} &  \text { if } i \ge 2 \text { and } j \text { is even,} \\ 0 &  \text { else.} \end{array}\right. } \qquad \text {for } {\left\{ \begin{array}{ll} i = 1, \dots , t + 1 \\ j = 1, \dots , 2t + 2 \end{array}\right. } \end{aligned}$$

By Equation (2), we can observe

4 $$\begin{aligned} \tilde{B}_{i,j}&= 0 \text { if } i \notin \left\{ \tfrac{j}{2}, \tfrac{j}{2} + 1 \right\} . \end{aligned}$$

So far, we reformulated $-e_1r + D_k z = e_t$ as an equivalent system $e_1 r + {\tilde{S} \atopwithdelims ()\tilde{B}} \tilde{z} = e_{\tilde{t}}$ for $\tilde{t}=2t+3$. Finally, we can permute the rows of ${\tilde{S} \atopwithdelims ()\tilde{B}}$, alternatingly taking a row of $\tilde{S}$ and $\tilde{B}$, and obtain the systemwhere $\star \in \{-1,0,1,2\}$ are the corresponding entries of $\tilde{B}$ respectively. We define the corresponding constraint matrix *T* as

$$ T_{i,j} \mathrel {\mathop :}={\left\{ \begin{array}{ll} \tilde{S}_{(i+1)/2,j} &  i \text { odd}, \\ \tilde{B}_{i/2,j} &  i \text { even}, \end{array}\right. } $$

and has dimension $(2t + 3) \times (2t + 2)$. It remains to verify that *T* is tri-diagonal. Depending on $i \mod 2$ we obtain by Conditions (3) and (4)

$$\begin{aligned} T_{i,j}&= \tilde{S}_{(i+1)/2,j} = 0  &   \text {if } i \notin \{j,j+1,j+2\}, \\ T_{i,j}&= \tilde{B}_{i/2,j} = 0  &   \text {if } i \notin \{j, j + 2\}. \\ \end{aligned}$$

This way, we obtain a tri-diagonal matrix $T_k \in \mathbb {Z}^{(2t + 3) \times (2t+2)}$ for every block ${c^{(k)} \atopwithdelims ()B}$, and are left with the system5that is equivalent to (2-stage stochastic IP).

Choose parameters $1 \le \varkappa \le t + 2$ and $\tau -1 \ge 1$ such that $2(\varkappa +1)^{\tau -1} \ge 2t + 4 > 2(\varkappa +1)^{\tau -2}$, implying $2(\varkappa +1)^{o(\tau -1)} \in o(t) = o(\log _2(N))$. After possibly adding some zero rows and columns, we apply Corollary 1, and regard each $T_k$ as a multi-stage matrix with $\tau$ stages and stage sizes $2\varkappa \cdot \textbf{1}$. In the worst case, the number of rows and columns are squared in the transformed matrix due to the padding needed for Corollary 1. Using the global variable as a first stage, we obtain a multi-stage integer program with $\tau$ stages and stage sizes $\sigma \mathrel {\mathop :}=(1,2\varkappa ,\dots ,2\varkappa )$ that is equivalent to (2-stage stochastic IP). Furthermore, all entries are still bounded by 2 and the dimensions are at most quadratic in the dimensions of (2-stage stochastic IP).

Denote by $g = 2^{\prod _{i=1}^\tau (\sigma _i + 1)}$ and $h=2^{o(\log _2(N))}$. It follows from the above, if there is an algorithm solving every multi-stage IP in time

$$ 2^{g^{o(1)}} |I |^{O(1)} \le 2^{h} |I |^{O(1)} \le 2^{o(N)} |I |^{O(1)}, $$

we could solve 3-Sat in time $2^{o(N)} |I |^{O(1)}$, contradicting ETH [17]. $\square$

## Tree-fold integer programming

In this section, we consider tree-fold IPs, whose constraint matrices are the transpose of multi-stage matrices. Our results can be viewed as a refinement of [29, Theorem 4]; while [29] only considers a single parameter (namely the *tree-depth*, discussed in Section 5), we take more aspects of the structure of the matrix into account. For example, as one case of our lower bound, we obtain the currently best known lower bound for the special class of *n*-fold IPs, i.e., tree-fold IPs with only two levels.

We reduced from the Subset Sum problem. There, we are given numbers $a_1,\dots ,a_n,b \in \mathbb {Z}_{\ge 0}$, and the task is to decide whether there exists a vector $x \in \{0,1\}^n$ satisfying $\sum _{i=1}^n a_i x_i = b$. Since all integers are non-negative, we can compare each $a_i$ to *b* beforehand, and henceforth assume $0 \le a_i < b$ for all *i*.

### Lemma 2

([29, Lemma 12]) There is no algorithm solving every instance *I* with $a_1,\dots ,a_n,b$ for Subset Sum in time $2^{o( n + \log _2(b) )}$ unless ETH fails.

### Theorem 3

(A lower bound for tree-fold IPs) For every number of levels $\tau \ge 1$, there is a family of tree-fold IPs with $\tau$ levels, constant level sizes, and entries bounded by a constant, such that there is no algorithm solving every instance *I* of this family of tree-fold IPs in time $2^{2^{2^{o(\tau )}}} |I|^{O(1)}$ unless ETH fails.

As a corollary, we obtain the following lower bound for *n*-fold IPs.

### Corollary 3

(A lower bound for *n*-fold IPs) There is no algorithm solving every *n*-fold IP instance *I* with level sizes $(\sigma _1,\sigma _2)$ in time $2^{o(\sigma _1 \sigma _2)} |I|^{O(1)}$ unless ETH fails.

### Proof idea 3

(of Theorem 3) We start with an instance of the Subset Sum problem. The goal is to first model this problem as an appropriate *n*-fold IP. Then, observing that the second level block-diagonal matrices are bi-diagonal allows us to apply Corollary 1, yielding the desired algorithm.

Let us thus focus on sketching how to obtain the *n*-fold matrix. We start with the straight-forward interpretation of Subset Sum as an integer program, that is, $\{x \in \{0,1\}^n : \sum _{i=1}^n a_i x_i = b\}$. We could directly apply the standard encoding as in Theorem 2 to lower the size of the entries. However, we can improve upon this by using a double encoding of the large entries: First, we encode them to the base of $\Delta$ (largest number $a_i, i \in [n]$) with $\Delta ^{\sigma _1} \ge b > \Delta ^{\sigma _1-1}$ (recall that $\sigma _1$ is the size of the first level, a parameter which we can chose between $1 \le \sigma _1 \le \lceil \log _2(b) \rceil$). Replacing a large entry with a matrix of its encoding is standard and done e.g. in [17]. We use the transposed construction though, and then, for a smaller basis, for example, the one from [17]. With this construction, the statement immediately follows.

### Proof of Theorem 3

For $\tau = 1$, we have arbitrary IPs, and the statement follows by [29] Theorem 1.

Let $a_1,\dots ,a_n, b \in \mathbb {Z}_{\ge 0}$ be a Subset Sum instance. Choose a number $1 \le \sigma _1 \le \lceil \log _2(b) \rceil$ and let $\Delta \in \mathbb {Z}_{\ge 2}$ such that $\Delta ^{\sigma _1} \ge b > \Delta ^{\sigma _1-1}$, with $\Delta = b$ if $\sigma _1=1$. In both cases, we have

6 $$\begin{aligned} \sigma _1 \log _2(\Delta )&\le 2 \log _2 (b) \le 2 \sigma _1 \log _2(\Delta ). \end{aligned}$$

Let $\lambda = (1,\Delta ,\Delta ^2,\dots ,\Delta ^{\sigma _1-1})^\intercal$ and let $c_i \in \{0,\dots ,\Delta -1\}^{\sigma _1}$ be the $\Delta$-encoding of $a_i$, i.e., $a_i = \lambda ^\intercal c_i$. Observe that $a^\intercal x = b$ has a solution $x \in \mathbb {Z}_{\ge 0}^n$ if and only if the system 
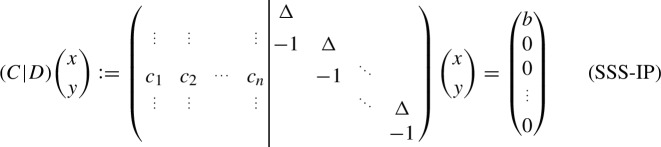
 has a solution ${x \atopwithdelims ()y} \in \mathbb {Z}_{\ge 0}^{n + \lceil \log _2(\Delta )\rceil }$. The “if”-direction is proven since $\lambda ^\intercal (C,D) = (a^\intercal ,0)$; we reduced from the Subset Sum problem. The “only-if”-direction follows as a solution *x* of Subset Sum fixes the values of *y* in the IP.

Let $t \mathrel {\mathop :}=\lceil \log _2 \Delta \rceil$ and fix a column $c_k$ of *C*. We can express each entry of $c_k$ in its binary representation. That is, let $z = (1,2,4,\dots ,2^{t-1})^\intercal \in \mathbb {Z}^{t}$ and let $C_k \in \{0,1\}^{\sigma _1 \times t}$ be the unique matrix subject to $C_k z = c_k$. Similarly, for the *k*-th column $d_k$ of *D*, there is a unique matrix $D_k \in \{-1,0,1\}^{\sigma _1 \times t}$ subject to $d_k = D_k z$ for $k = 1, 2, \dots , s$ with $s = \lceil \log _2 (\Delta +1) \rceil$.

Again, let the encoding matrix be the matrix7and observe that the system $E_t x = 0$ with $x \in \mathbb {Z}^{t}$ and $0 \le x < 2^t$ only has the two solutions $\{0,z\}$. Therefore, the Subset Sum instance is a yes-instance if and only if the system 
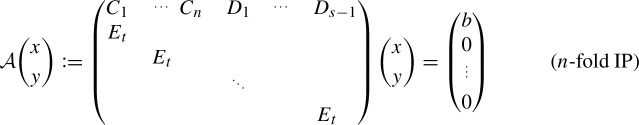
 has a solution satisfying $0 \le x < 2^t$ as we only encoded the entries and thus the solution to base 2 of SSS-IP and bounded the size of a solution. We reduce from the Subset Sum problem. The “only-if”-direction follows as a solution *x* of Subset Sum fixes the values of *y* in the IP.

So far, the constructed IP is an *n*-fold IP with $\sigma _1 \in \Theta (\tfrac{\log _2(b)}{\log _2(\Delta )})$ rows in the top blocks, $t \in \Theta (\log _2 (\Delta ))$ columns per block, and $\hat{\sigma }_2 = t-1$ rows in the diagonal blocks. We have $n+\sigma _1$ blocks in total, and $\Vert \mathcal {A}\Vert _{\infty } \le 2$, hence the size of the constructed instance is polynomial in the size of the Subset Sum instance. It follows that if we could solve it in time

$$ 2^{o(\sigma _1 \hat{\sigma }_2)}|I|^{O(1)} \le 2^{o(\sigma _1 \log _2(\Delta ))}|I|^{O(1)} {\mathop {\le }\limits ^{(6)}} 2^{ o ( \log _2 (b) ) }|I|^{O(1)}, $$

then this would contradict Lemma 2, finishing the proof for $\tau = 2$ (Corollary 3).

To prove the statement for $\tau \ge 3$, we continue the construction. Choose $\tau$ such that $2 \le \tau -1 \le \lceil \log _2(t) \rceil$ and $s \ge 1$ such that $(s+1)^{\tau -1} \ge t > s^{\tau -1}$. Furthermore, let $\ell \ge 0$ be such that $(s+1)^{\tau -1-\ell }s^{\ell } \ge t > (s+1)^{\tau -\ell -2}s^{\ell +1}$. Observe that if $s=1$, then $\ell = 0$, since $\tau -1 \le \lceil \log _2(t) \rceil$. Let $(\sigma _2,\dots ,\sigma _{\tau }) \in \{s-1,s\}^{\tau -1}$ have $\tau - 1 - \ell$ entries *s* and $\ell$ entries $s-1$. This careful construction of $\sigma$ allows us to estimate

8 $$\begin{aligned} \prod _{i=2}^{\tau } (\sigma _i + 1) = (s+1)^{\tau -1-\ell }s^{\ell }&= \frac{s+1}{s} (s+1)^{\tau -\ell -2}s^{\ell +1} < 2t. \end{aligned}$$

Since $E_t^\intercal$ is bi-diagonal, we can extend $E_t$ with zeros to a tree-fold matrix with $\tau -1$ levels and level sizes $(\sigma _2,\dots ,\sigma _{\tau })$ due to Corollary 2. In total, the matrix $\mathcal {A}$ is a tree-fold matrix with $\tau$ levels and level sizes $\sigma$, and has the same encoding length as the instance we reduced from. If there is an algorithm solving every such tree-fold IP in time

$$ 2^{o( (\sigma _1+1) \prod _{i=2}^{\tau } (\sigma _i+1) )} {\mathop {\le }\limits ^{(8)}} 2^{o( 2\sigma _1 2t )} \le 2^{o( 8 \sigma _1 \log _2(\Delta ) )} {\mathop {\le }\limits ^{(6)}} 2^{o( 16 \log _2(b))}, $$

this would again contradict Lemma 2. $\square$

## Lower bounds on the Graver basis

### Definition 5

(*Graver Basis*) Let $A \in \mathbb {Z}^{m \times n}$. The Graver basis $\mathcal {G}(A)$ of *A* is the set of all vectors $z \in \ker (A) \cap \mathbb {Z}^n\setminus {\{(0, \dots , 0)^\top \}}$ that cannot be written as $z = x + y$ with $x,y \in \ker (A) \cap \mathbb {Z}^n\setminus {\{(0, \dots , 0)^\top \}}$ satisfying $x_iy_i \ge 0$ for all $i \in [m]$. Let *K* be some norm. We define $g_{K} (A) = \max _{g \in \mathcal {G}(A)} \Vert g \Vert _{K}$.

Our previous results state that, assuming ETH, there is no algorithm for multi-stage or tree-fold IPs that solves *every* instance within a certain time threshold. We now show further evidence orthogonal to ETH. All known algorithms for block-structured IPs have $g_\infty (A)$ or $g_1(A)$ in the running time complexity [6]. Thus, if there is no fundamentally different algorithm for those problems, lower bounding $g_\infty (A)$ and $g_1(A)$ lower bounds the complexity of those problems. We show that the instances we constructed, in particular, the encoding matrix $E_t$ (see (2-stage stochastic IP) and (*n*-fold IP)), indeed have large $g_\infty (E_t)$ and $g_1(E_t)$, respectively.

### Lemma 3

Let $t \ge 2$, $\Delta \in \mathbb {Z}_{\ge 2}$. The encoding matrixsatisfies $g_{\infty }(E_t(\Delta )) \ge \Delta ^{t-1}$ and $g_1(E_t(\Delta )) \ge \tfrac{\Delta ^t - 1}{\Delta - 1}$.

### Proof

The matrix $E_t(\Delta )$ has full row rank. Therefore, its kernel has rank $t-(t-1) = 1$. Since $z^\intercal \mathrel {\mathop :}=(1,\Delta ,\dots ,\Delta ^{t-1}) \in \ker (E_t)$, every element in $\ker (E_t) \cap \mathbb {Z}^{t}$ is an integer multiple of *z*. Thus, $\{z, -z\}$ is the Graver basis of $E_t(\Delta )$. $\square$

In [32], Lee et al. show that $g_1(A) \le O(m^3 \Vert A \Vert _\infty ^m$) for any matrix $A \in \mathbb {Z}^{m \times n}$, so $E_t$ is the worst case up to a factor $m^3$. As an immediate consequence of Theorem 1 and Lemma 1, we get:

### Theorem 4

Let $\tau \in \mathbb {Z}_{\ge 1}$, $\sigma \in \mathbb {Z}^{\tau }_{\ge 1}$, and define $S \mathrel {\mathop :}=\prod _{i=1}^\tau (\sigma _i + 1)$.

1. There is a multi-stage matrix *A* with $\tau$ stages and stage sizes $\sigma$ that satisfies $g_{\infty }(A) \ge \Vert A \Vert _{\infty }^{S - 2}$.
2. There is a tree-fold matrix *A* with $\tau$ levels and level sizes $\sigma$ that satisfies $g_{1}(A) \ge \Vert A \Vert _{\infty }^{S - 1}$.

### Proof

For the first claim, observe that the matrix $E_{S - 1}(\Delta )$ satisfies $g_{\infty }(E_{S - 1}(\Delta )) \ge \Delta ^{S-2}$. If *A* arises from $E_{S - 1}(\Delta )$ by adding a zero row in the top and the bottom, we immediately obtain $g_{\infty }(A) \ge \Delta ^{S-2}$, and can apply Corollary 1.

For the second claim, we can apply Corollary 2 to the matrix $E_{S}(\Delta )$. $\square$

## Beyond block structure: tree-depth

There is the more general notion of primal and dual *tree-depth* of *A* to capture the above classes of block-structured IPs. This section provides a brief introduction and states our results in terms of these parameters.

The *primal graph* *G* of *A* has the columns of *A* as a vertex set, and an edge between two columns *a*, *b* if they share a non-zero entry, i.e., there is an index *i* with $a_ib_i \ne 0$. A *td-decomposition* of *G* is an arborescence *T* with $V(T) = V(G)$ such that for any edge $\{u,v\} \in E(G)$ there either is a (*u*, *v*)-path in *T* or a (*v*, *u*)-path. A td-decomposition of *G* with minimum height is a minimum td-decomposition. The primal tree-depth ${{\,\textrm{td}\,}}_P(A)$ of *A* is the maximum number of vertices on any path in a minimum td-decomposition of *G*. The *dual graph* and tree-depth are the primal graph and tree-depth of $A^\intercal$.

If *A* is a multi-stage matrix with $\tau$ stages and stage sizes $\sigma$, we can construct a primal td-decomposition: Start with a path through the first $\sigma _1$ columns. Since the rest of the matrix decomposes into blocks, there are no edges between any two blocks, and any edge inducing a path either is from one of the first $\sigma _1$ columns to a block, or within a block. Thus, we can recurse on the blocks and append another path of $\sigma _2$ columns to column $\sigma _1$. This way, we obtain a td-decomposition of height $\sum _{i=1}^\tau \sigma _i - 1$, and get:

### Lemma 4

1. Let *A* be multi-stage matrix with $\tau$ stages and stage sizes $\sigma$. Then ${{\,\textrm{td}\,}}_P(A) \le \sum _{i=1}^\tau \sigma _i$.
2. Let *A* be tree-fold with $\tau$ levels and level sizes $\sigma$. Then ${{\,\textrm{td}\,}}_D(A) \le \sum _{i=1}^\tau \sigma _i$.

Using the constructions of Theorems 2 and 3, we can chose $\sigma \le 2 \cdot \textbf{1}$ and hence, ${{\,\textrm{td}\,}}_P(\mathcal {A}) \le 2 \tau$, ${{\,\textrm{td}\,}}_D(\mathcal {A}) \le 2\tau$ respectively. We obtain the following corollary. While the second point was already proven in [29], the first point is a new consequence.

### Corollary 4

There is no algorithm solving every IP instance *I* in time $2^{2^{2^{o( {{\,\textrm{td}\,}}_P(A))}}} |I|^{O(1)}$, nor in time $2^{o (2^{{{\,\textrm{td}\,}}_D(A)})} |I|^{O(1)}$ unless ETH fails.

## Footnotes

1 There exist other, similar concepts of parameterized diagonal matrices where e.g., $a_{i,j} = 0$ for $j < i-c_1$ or $j > i+c_2$ for some constants $c_1, c_2$ [11, 13].
